# Alzheimer’s, Parkinson’s, Frontotemporal Lobar Degeneration, and Amyotrophic Lateral Sclerosis Start in Pediatric Ages: Ultrafine Particulate Matter and Industrial Nanoparticles Are Key in the Early-Onset Neurodegeneration: Time to Invest in Preventive Medicine

**DOI:** 10.3390/toxics13030178

**Published:** 2025-02-28

**Authors:** Lilian Calderón-Garcidueñas, Angélica González-Maciel, Rafael Reynoso-Robles, Fredy Rubén Cejudo-Ruiz, Héctor G. Silva-Pereyra, Andrew Gorzalski, Ricardo Torres-Jardón

**Affiliations:** 1 College of Health, The University of Montana, 287 Skaggs Building, 32 Campus Drive, Missoula, MT 59812, USA; 2 Instituto Nacional de Pediatría, Mexico City 04530, Mexico; agonzalezmaciel@yahoo.com (A.G.-M.); reynosoraf@yahoo.com (R.R.-R.); 3 Instituto de Geofísica, Universidad Nacional Autónoma de México, Mexico City 04510, Mexico; ruben@igeofisica.unam.mx; 4 Instituto Potosino de Investigación Científica y Tecnológica AC, San Luis Potosi 78216, Mexico; hector.silva@ipicyt.edu.mx; 5 Nevada Genomics Center, University of Nevada at Reno, Reno, NV 89556, USA; andrewg@unr.edu; 6 Instituto de Ciencias de la Atmósfera y Cambio Climático, Universidad Nacional Autónoma de México, Mexico City 04510, Mexico; rtorres@unam.mx

**Keywords:** pediatric neurodegenerative diseases, fine particulate matter, ultrafine PM, nanoparticles, Alzheimer, Parkinson, children, TDP-43 pathology, magnetic particles, fires, dementia

## Abstract

Billions of people are exposed to fine particulate matter (PM_2.5_) levels above the USEPA’s annual standard of 9 μg/m^3^. Common emission sources are anthropogenic, producing complex aerosolized toxins. Ultrafine particulate matter (UFPM) and industrial nanoparticles (NPs) have major detrimental effects on the brain, but the USA does not measure UFPM on a routine basis. This review focuses on the development and progression of common neurodegenerative diseases, as diagnosed through neuropathology, among young residents in Metropolitan Mexico City (MMC). MMC is one of the most polluted megacities in the world, with a population of 22 million residents, many of whom are unaware of the brain effects caused by their polluted atmosphere. Fatal neurodegenerative diseases (such as Alzheimer’s and Parkinson’s) that begin in childhood in populations living in air polluted environments are preventable. We conclude that UFPM/NPs are capable of disrupting neural homeostasis and give rise to relentless neurodegenerative processes throughout the entire life of the highly exposed population in MMC. The paradigm of reaching old age to have neurodegeneration is no longer supported. Neurodegenerative changes start early in pediatric ages and are irreversible. It is time to invest in preventive medicine.

## 1. Introduction

Air pollution is a serious health threat for millions of citizens [1]. Billions of people are exposed to levels of criteria air pollutants above the current USEPA standards, including carbon monoxide (CO), lead (Pb), ground-level ozone (O_3_), particulate matter (PM), nitrogen dioxide (NO_2_), and sulfur dioxide (SO_2_) [1]. Causes of mortality and morbidity associated with air pollutants include cardiovascular diseases, stroke, chronic obstructive pulmonary disease, cancer, and pneumonia [2,3,4,5,6,7]. Neurodegenerative diseases and their strong association with air pollutants in children and young adults are critical [8,9,10,11,12,13,14,15,16,17].

**Figure 1:**
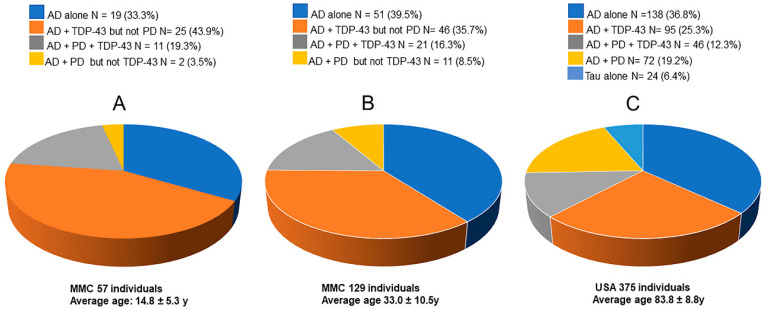
(**A**) Alzheimer’s disease (AD) (hyperphosphorylated tau, beta amyloid plaques), Parkinson’s disease (PD) (α-synuclein), and transactive response DNA-binding protein 43 (TDP-43) pathology overlap in forensic autopsies of 14.8 ± 5.3-year-olds in MMC. (**B**) In MMC, 33.0 ± 10.5-year-olds exhibit the same pathology as children and teens. (**C**) Unimpaired and cognitively impaired older US residents show similar quadruple proteinopathies [18] as young people in MMC. Data from Calderón-Garcidueñas et al. [9].

There is a paradigm shift regarding fatal neurodegenerative diseases: the neuropathology of Alzheimer’s disease (AD), Parkinson’s disease (PD), frontotemporal lobar degeneration (FTLD), and amyotrophic lateral sclerosis (ALS) starts in pediatric ages (Figure 1) [8,9,10,11,12,13,14,15,16,17]. Figure 1 shows the neuropathological hallmarks of AD, PD, and TDP-43 pathology in children and young adults in Metropolitan Mexico City (MMC) versus elderly people in a community-based cohort at the University of Kentucky: the neurodegenerative diseases are the same [18]. UFPM and NPs resulting from combustion processes (including ubiquitous anthropogenic combustion emissions, wildfires, and domestic use of wood/pellets/stoves/ fireplaces), friction-derived nanoparticles, and industrial sources are at the core of the problem [5,7].

Metropolitan Mexico City (MMC) is a polluted megacity with a population of 22 million. Residents are exposed to daily high concentrations of PM_2.5_ above the USEPA annual standard, mainly from combustion emissions (diesel heavy vehicles included), consistent with other megacities across the world [19,20,21,22,23,24,25,26,27,28,29,30,31,32,33].

Overlapping common and uncommon fatal neurodegenerative diseases have been described in MMC residents as young as 11 months [8]. The impact of sustained and high concentrations of PM_2.5_ from conception to death has major detrimental effects on the central nervous system, heart, and other organs [10,15,17].

The studies highlighted in this review detail a paradigm shift regarding fatal neurodegenerative diseases: the neuropathology of Alzheimer’s disease (AD), Parkinson’s disease (PD), frontotemporal lobar degeneration (FTLD), and amyotrophic lateral sclerosis (ALS) starts in pediatric ages (Figure 1) [8,9,10,11,12,13,14,15,16,17]. This review will present our published experience collected in a major megacity, along with relevant contributions from three key research groups: nanotoxicologists looking at the neural effects of NPs, investigators working on creating nanosized structures capable of reaching the brain for therapeutic/diagnostic goals, and physicists demonstrating that electromagnetic fields are causing motion behaviors in the magnetic UFPM/NPs identified in brain tissue.

Neural organelles exhibit an array of nanosized (≤100 nm) particulate matter, composed of metals, metalloids, and natural elements from anthropogenic, industrial, and natural sources (volcanic eruptions and fires) [10,11,12,13,14,15,16]. These findings correlate with overlapping AD, PD, FTLD, and ALS neuropathological hallmarks in children and young adults [8,9,10,11,12,13,14,15,16,17]. Sources such as fireplaces to keep your home warm, the use of pellets containing harmful metals, and complex indoor emission exposures are also relevant to brain pathology [34,35].

Ignoring the data of MMC children and the overlapping quadruple neuropathology documented in 99% of residents is not beneficial to the millions of citizens exposed to pollutants, unaware of the harmful impacts on their brain and the fact that the brain damage starts in utero [8,10]. Remarkably, from 203 consecutive forensic autopsies in subjects ≤40 years old in MMC, only one 21-year-old female had no evidence of neurodegeneration [8]. The 203-autopsy sample included subjects from all areas of MMC and varying socioeconomic statuses [8].

The neural toxicity of UFPM/NPs depends on the type of cell and other ubiquitous key variables in 21st-century environments, including massive exposure to electromagnetic fields and earphone/smartphone-embedded magnetism, which is potentially capable of moving magnetic particles located in critical neural organelles, as we have shown in MMC residents’ brains [16,36,37,38,39,40,41,42,43,44,45,46,47].

These are our main premises: Fatal neurodegenerative diseases that develop from childhood in populations living in air polluted environments are preventable. The notion that neurodegeneration only occurs in old age is no longer supported. Neurodegenerative changes start early in pediatric ages and are irreversible.

## 2. Air Pollution

### 2.1. Air Pollution in Urban Environments and Metropolitan Mexico City as an Example of Complex Air Pollution and Chronic Neurotoxic Exposure

Air pollution, including fine particulate matter (PM_2.5_) pollution, is a serious health problem in MMC and across the globe [19,34,35].

MMC covers ~7585 km^2^ in an elevated 2240-m basin, with mountains on three sides. MMC residents are exposed to microscopic particulate matter (PM) solids of different sizes, aerosols, and chemicals. For this review, we will focus on PM_2.5_ (particles < 2.5 µm), ultrafine particulate matter (UFPM), and nanoparticles (NPs) with an aerodynamic diameter (<0.1 µm). PM differs in chemical composition, size, shape, morphology, and air lifetime, depending on its origin, i.e., primary or secondary [7,19,20,21,22,23,24,25,26,27,28,29,30,31,32,33].

In addition to secondary PM_2.5_, atmospheric concentrations include those emitted directly from anthropogenic sources, such as incomplete combustion processes from the transportation sector—around 43%, with heavy diesel vehicles having the highest contribution [19]. Biomass combustion, industrial processes, and non-exhaust sources, such as the wearing down of brake pads, clutches, tires, and road surfaces, contribute ~36%. Micrometer-sized particles are important sources of metals, including cooper (Cu), zinc (Zn), iron (Fe), and aluminum (Al) [22].

MMC residents have been exposed in the last three decades to PM_2.5_ levels higher than the national and international air quality guidelines [9,19]. Episodes of very high levels of PM_2.5_ in MMC are typically associated with the transport of dense smoke plumes from fires [21]. The typical composition of urban suspended particles is dominated by the presence of organic matter, i.e., 50% of the total mass. Particles formed within the atmosphere through nucleation, condensation, and/or chemical reactions of gas-phase species constitute secondary PM. These secondary particles dominate the PM_2.5_ composition between mid-morning and noon, when solar radiation is intense [19]. On the other hand, PM _0.1_ represents around 70% of PM_2.5_. The most abundant species in PM_0_._1_ in MMC include black carbon (BC), nitrate (NO_3_^−^), sulfate (SO_4_^2−^), and ammonium (NH_4_^+^). Particles in the size range of 10 and 400 nm and particle number concentrations between 10,000 and 40,000 # cm^−3^ have a strong relationship with vehicle emissions [30].

PM_2.5_ levels are considered a proxy for PM pollution; however, the fraction we are interested in is ultrafine PM/NPs with a negligible mass, making them difficult to measure. UFPM is quantified based on the number of particles and consists of volatile and semi-volatile carbon-bearing phases, as well as solid combustion vehicle-derived particles containing highly reactive transition metals (i.e., Fe, Cu, Mn, Ti, Cr, Ni, V, Pb, and Zn) [26,27,28,29]. The metal concentrations in UFPM follow this order: Fe > Zn > Pb > Mn > Hg > Sn > Ni > Cr > Ti > V > Ag [29]. MMC operates an old fleet of heavy diesel vehicles that is responsible for the high level of UFPM emissions.

**Figure 2:**
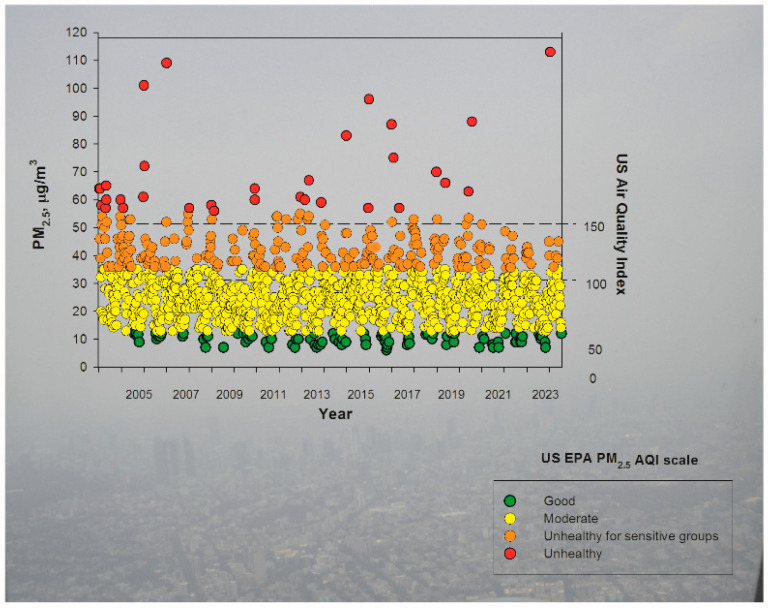
Trend of maximum mean 24-h PM_2.5_ concentrations for five representative MMC monitoring stations from 2004 to July 2024 and their comparison with the respective USEPA AQI categories and index values. The graph is overlaid on an aerial picture of MCM taken by LCG, during a PM_2.5_ episode on 13 December 2024. Pollution data obtained from: http://www.aire.cdmx.gob.mx/aire/default.php (accessed on 8 January 2025).

MMC residents have been chronically exposed to PM_2.5_ concentrations above the current annual USEPA standard (9 µg/m^3^) (Figure 2). The COVID-19 period was not an exception for high PM_2.5_ exposures, despite the fact that private vehicles, for the most part, were not allowed to circulate. Toxic emissions are coming from more than 60,000 industries (1000 highly polluting), around 100,000 heavy diesel passenger and cargo trucks, and over 1 million motorcycles not subjected to any regulatory standards.

UFPM is at the core of this work, with multiple studies showing that the distribution of UFPM/NPs in MMC varies within the city, with high concentrations related to heavy diesel vehicles, high-traffic areas, and slow-speed hot spots (30–33).

### 2.2. Ultrafine Particulate Matter and Industrial Nanoparticle Sources, Portals of Entry, Magnetic Motion Behavior, and Ubiquitous Exposure to Electromagnetic Fields

UFPM, industrial NPs, and electromagnetic fields are ubiquitous around the world. The portals of entry for UFPM/NPs—i.e., upper and lower respiratory tract, gastrointestinal tract, placenta, skin, and mucosae—as well as their sources and composition have been described by our laboratory. The targeted areas are associated with neuropsychiatric burden, sleep disorders, audition, olfaction, gait, and balance complaints in seemingly healthy residents [8,9,10,11,12,13,14,15,16]. In addition, UFPM/NPs have been described in the atrioventricular conduction axis in association with ultrastructural damage and anthropogenic, industrial, and E-waste NPs [15].

Key to the early development of neurodegeneration in urban young residents are Fe single-domain NPs composed of magnetite and maghemite, which are capable of movement when exposed to electromagnetic fields [16]. The health impact of weak radiofrequency electromagnetic fields (RF-EMFs) is of concern given the proximity of sources that transmit these waves, including phones, computers, Bluetooth devices, and power lines. Radiofrequency (RF) EMFs and extremely low-frequency (ELF) MFs have been classified as possibly carcinogenic to humans (Group 2B) by the International Agency for Research on Cancer (IARC) [36]. The frequency band at 6.78 MHz is a designated Industrial, Scientific, and Medical (ISM) band [37], and we have no limits on electromagnetic emissions from ISM equipment at this frequency. RF-EMF radiation is unavoidable, we have no protection or clear safety standards; thus, modification of the existing guidelines, standards, and regulations is needed [38,39,40,41,42,43,44,45,46,47].

Based on the current literature, it is biologically plausible that the motion of highly toxic, oxidative, magnetic NPs under magnetic fields [16] could result in magnetic hyperthermia, diffusion, convection, residual magnetization, and electromagnetic drift [48,49,50,51,52,53,54].

**Figure 3:**
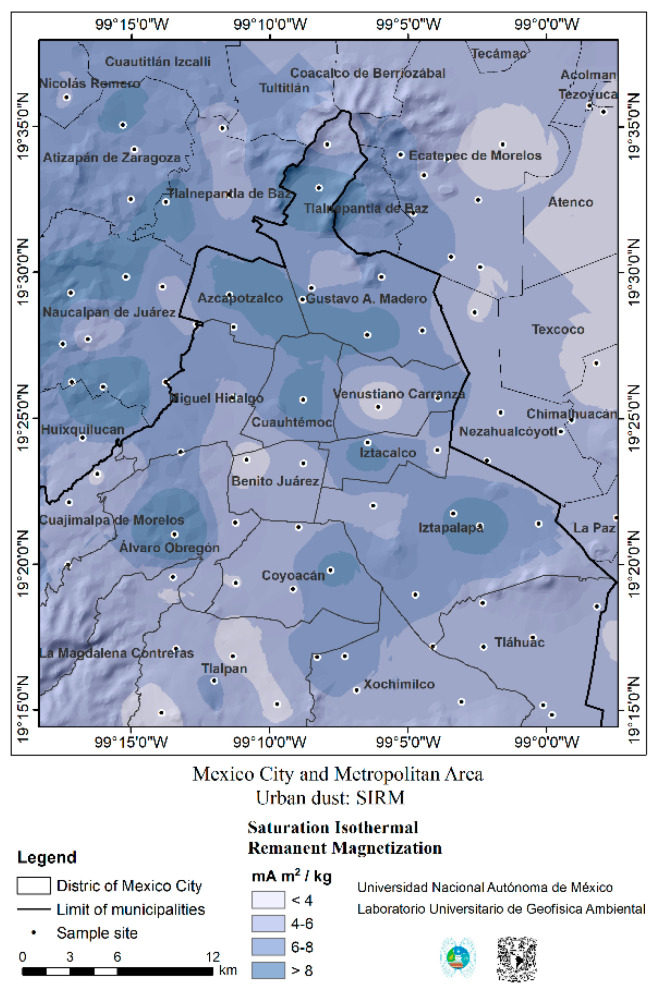
Magnetic susceptibility (X_lf_) map. Spatial distribution of magnetic susceptibility obtained from road dust samples in MMC. X_lf_ is an indicator of the accumulation of magnetic minerals. MMC showed four areas with different concentrations of magnetic minerals. Two sites to the northeast exhibited low concentrations (X_lf_ < 3 µm^3^ kg^−1^), while medium-concentration zones (X_lf_ between 3 and 6 µm^3^ kg^−1^) were located in the northeast, east, and south of MMC. In sharp contrast, high concentrations (X_lf_ between 6 and 9 µm^3^ kg^−1^) were found in the NW and SE directions, the most urbanized and populated regions. Areas with very high concentrations of magnetic materials (X_lf_ > 8 µm^3^ kg^−1^) were located in the eastern (Ixtapalapa), north-central (Azcapotzalco, Gustavo A. Madero), and western (Huixquilucan) areas of MMC.

The distribution of magnetic UFPM/NPs in urban dust in MMC is shown in Figure 3. The map shows areas with high magnetic dust concentrations (30–33). We expect the greatest extent of brain damage among these residents. Interestingly, some of the most polluted areas in MMC have the highest levels of criminal violence [55].

The presence of magnetic UFPM/NPs in the brains of all MMC residents is very concerning, as these predominantly iron-based nanoparticles (FeNPs) are highly reactive and produce extensive oxidative stress. Their effects related to their magnetophoresis properties and exposure to electromagnetic fields (40–54).

The cytotoxic effects of redox-active, magnetic UFPM/NPs on the brain include lung cell and endothelial damage, leading to the production of reactive oxygen species (ROS), lactate dehydrogenase, and apoptosis [51,52]. NPs could also cause damage to the neurovascular unit (NVU) [56] by altering vascular and neural ROS production, inducing magnetic hyperthermia, reducing lysosomal performance, and altering the intrinsic permeability spectra, depending on the distribution of local effective magnetic fields [57,58,59,60,61,62,63,64,65,66,67].

The aggregation and agglomeration of iron oxide NPs impact their magnetic and heating properties. Most importantly, single-domain NPs < 30 nm and protein–NPs interactions are potentially relevant to the cytotoxicity of FeNPs in targeted hubs [68,69,70,71,72]. NPs’ shape is also critical, i.e., spherical vs. rod-shaped NPs [73].

Authors have previously described the typical high-temperature magnetite nanospheres commonly found in MMC air and dust particulate matter pollution [74]. Zhao et al. [73] showed that rod-shaped NPs have a longer residence time in the gastrointestinal tract compared with spherical NPs. This is of utmost relevance to MMC residents, as long rod NPs have a significant capacity to overcome rapid clearance by the reticuloendothelial system and exhibit longer circulation in the blood than both short rods and spherical NPs. In addition, the degradation of long rod NPs takes longer, which the authors [73] attribute to their higher specific surface area.

**Figure 4:**
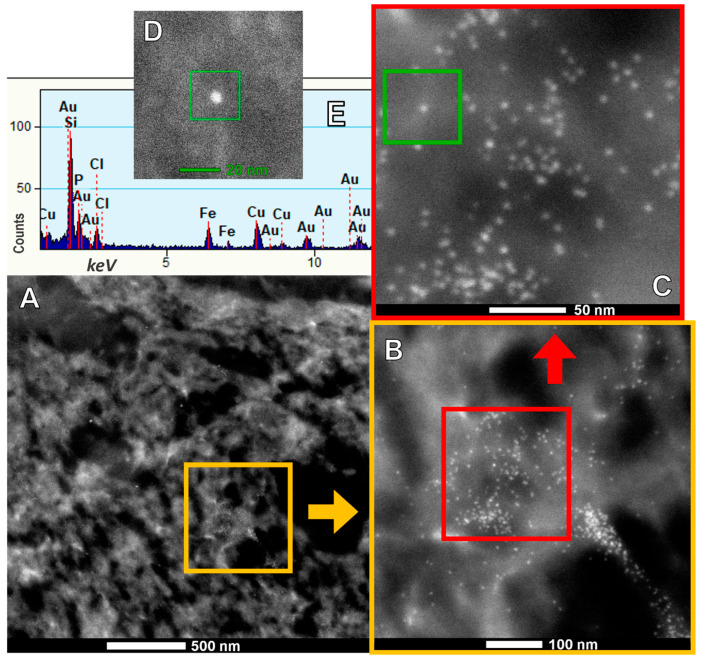
A series of micrographs obtained by an FEI TENAI F30 Transmission Electron Microscope (TEM), set to 100 kV in Z-contrast mode, are shown. FeNPs are extensively distributed in neural tissue. (**A**–**C**) Photographs with areas showing an increase in magnification, marked within the color squares; (**D**) is extracted from (**C**), indicating the region from which an EDX spectrum was obtained. The spectrum shown in (**E**) displays the recorded elements. Fe is present in the spherical single-domain NP analyzed (average size 7–10 nm). Au and Cu are the elements constituting the utilized grid.

Energy-dispersive X-ray spectrometry (EDX) studies have identified a range of metal, metalloid, and natural elements across neural and vascular organelles, such as Fe, Ni, Co, Ti, V, Hg, Cu, Zn, Cd, Al, Mg, Ag, Ce, La, Pr, W, Ca, Cl, K, Si, S, Na, and/or Br NPs in cortical and subcortical structures, including the brainstem and cerebellum (Figure 4) [13,14,15,16,74].

Magnetic UFPM and NPs exhibit mobility upon exposure to radiofrequency (RF) EMFs and extremely low-frequency (ELF) MFs, which is potentially hazardous for the brain.

**Figure 5:**
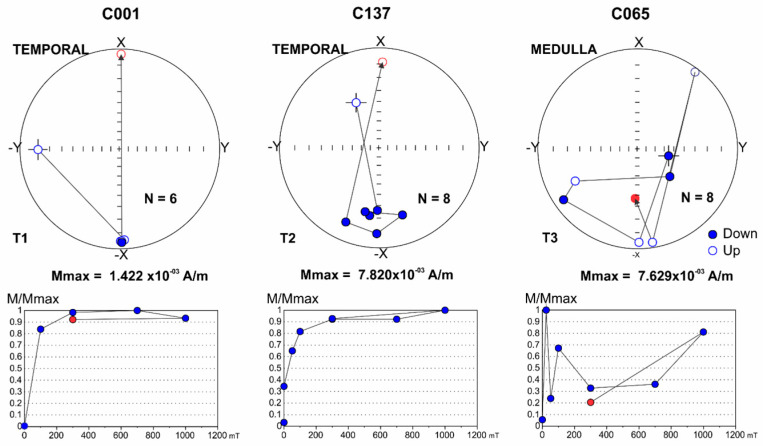
Representative orientation curves and plots during the acquisition of isothermal remanent magnetization (IRM) of low-coercivity minerals in brain samples.

Figure 5 illustrates the motion behavior of magnetic NPs in anatomical areas undergoing neurodegenerative changes. The motion behavior of UFPM/NPs is noticeably different according to the brain regions for each individual. The magnetic motion behavior of these NPs located in critical organelles could cause subcellular damage. T2 (small displacement) and T3 (significant displacement) motion, along with erratic behavior in mitochondria, ER, or heterochromatin, could result in irreversible damage (e.g., medulla of a 68-year-old man, CO65) [16].

Low-coercivity minerals are magnetized at 180° (-X direction) and maintain their orientation up to values of 1000 mT. This behavior occurs in materials that do not present movement within the sample and are classified as type T1 (temporal CO01 in a 22-year-old female). Minerals that show slight variations in position during MRI acquisition reflect changes in orientation around -X, between 15° and 30°, and are classified as type T2 (C137, temporal, 22-year-old female). Samples that do not maintain orientation in the -X direction indicate a strong displacement of the low-coercivity magnetic material and are classified as type T3 (CO65, medulla in a 68-year-old male) [16].

## 3. UFPM, NPs, Mitochondria, and Mitochondrial Dysfunction

**Figure 6:**
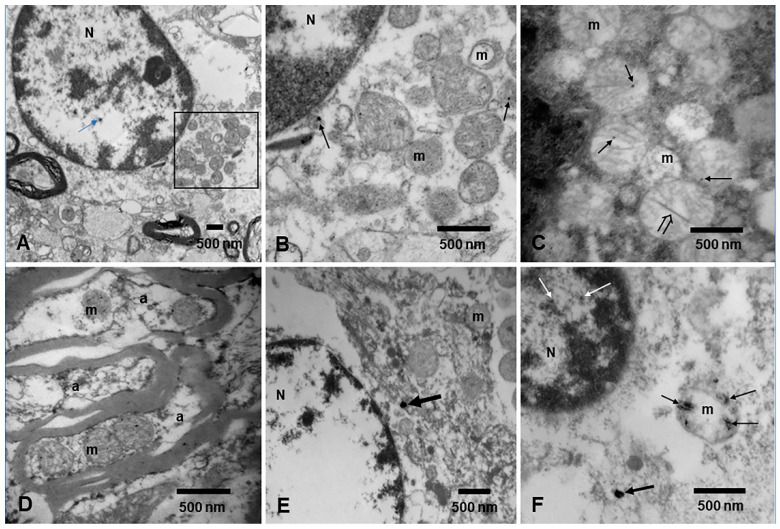
Mitochondria from neural tissues in MMC residents. (**A**) Olfactory bulb in a 13-year-old female with a cluster of mitochondria adjacent to the nucleus (blue arrow points to a NP) in an oligodendroglia. N is marking the nucleus. The black square is amplified in (**B**); EM ×15,000. (**B**) Mitochondria (m) with mark variation in size and abnormal cristae. The arrows point to larger, spherical anthropogenic nanoparticles. EM ×50,000. (**C**) Motor neuron in Brodmann 4 cortex in a 21-year-old male. Note the mitochondria (m) with abnormal cristae, all containing spherical NPs (short arrows). In contrast, the open arrow shows a long rod NP [68]. EM ×50,000. (**D**) Cerebellar vermis white matter in an 11-month-old. Mitochondria in myelinated axons (a) all show spherical NPs among abnormal cristae. EM ×50,000. (**E**) Caudate neuron with an NP in a small abnormal mitochondrion (arrow). EM ×30,000. (**F**) Fetal brain PCW 14 with NPs in both nucleus (N) (white arrows) and mitochondria (m) (short black arrows). Most mitochondrial NPs are short and long rods (thin black arrows). A spherical NP is marked with a thick black arrow. EM ×50,000.

UFPM and NPs are identified in mitochondria both in the neurovascular unit and in neuronal, glial, and microglial cells (Figure 6). Of significant importance, UFPM/NPs have been detected in the mitochondria of a fetal brain in utero [10]. Mitochondria are highly dynamic organelles, serving as primary ATP production sites and key regulators of apoptosis. Sustaining damage could result in mitochondrial dysfunction, including oxidative dysregulation, with neurodevelopmental consequences [10].

There is extensive distribution of mitochondrial combustion spherical magnetic NPs with an Fe-*L*_2,3_ EELS spectrum of magnetite [74] and typical T3 behavior of unstable magnetic NPs, which have the lowest polydispersity and the highest number of surface coating molecules [16]. These anthropogenic particles mostly contain magnetite (Fe_3_O_4_), maghemite (γFe_2_O_3_), titanomagnetite (Fe_3_O_4_-Fe_2_TiO_4_), and titanomaghemite (Fe_2_O_3_-FeTiO_3_) [16,74].

The physico-chemical interactions of FeNPs cause cytotoxic and radical responses [75,76], including Fe ion release and •OH radical production. The released free ions and free radicals interact with intracellular neural communication and affect regulation controlled by Ca^2+^ and redox signaling, leading to excitotoxicity, oxidative stress, and/or neuropathology [75,76].

Activation of the mitochondrial permeability transition (mPT) is a key mechanism linking mitochondrial calcium uptake and ROS production, and mPT gives rise to a dysregulated oxidative state with loss of GSH- and NADPH-dependent ROS detoxification [77,78]. It is possible that mPT, a phenomenon that abruptly causes the flux of low molecular weight solutes across the mostly impermeable inner mitochondrial membrane, results from the interaction of UFPM/NPs within the mitochondrial permeability transition pore (mPTP), a supramolecular entity assembled at the interface of the inner and outer mitochondrial membranes [79].

The presence of nanotunnels—thin double-membrane protrusions 40–200 nm in diameter which connect the matrices of non-adjacent mitochondria—allowing mitochondria to communicate at a distance with each other could also serve as NP innocent bystanders [80]. Nanotunnels emerge from the surface of immobilized mitochondria and/or from mitochondria with restricted motility [80].

Neuro- and nasal inflammation, mitochondrial responses, and neurodegeneration are recognized feedback loops [81,82,83,84,85,86]. Neuroinflammation starts 7 days after birth [84] and persists as MMC residents remain in the megacity [8,12,85]. Brain upregulation of cyclooxygenase-2, an inflammatory mediator; accumulation of Aβ42; increased genomic DNA apurinic/apyrimidinic sites, and nuclear factor-kappa B activation were documented very early in our studies, strongly suggesting that exposure to severe air pollution (NPs) is associated with brain inflammation and Aβ42 accumulation [84,85].

Mitochondrial damage-associated molecular patterns (mtDAMPs) are quickly recognized by microglial immune receptors, which induce neuroinflammation [86]. There is a consensus that mitochondrial dysfunction precedes neuroinflammation, and thus, the extensive deposition of magnetic NPs in mitochondrial matrices induces significant damage and dysfunction [86]. Lin et al. [86] described DAMPs that induce or aggravate neuroinflammation in neurodegenerative diseases, including mtDNA, mitochondrial unfolded protein response (mtUPR), mitochondrial reactive oxygen species (mtROS), adenosine triphosphate (ATP), transcription factor A mitochondria (TFAM), cardiolipin, cytochrome c, mitochondrial Ca^2+^, and iron. We strongly suggest adding magnetic UFPM/NPs [16] to the list of mtDAMPs inducers.

Regardless of age, impaired mitophagy leads to the accumulation of dysfunctional mitochondria, which release mitochondrial damage-associated molecular patterns (mtDAMPs) [87]. As described by Mishra et al. [87], mtDAMPs act as immune checkpoints, activating pattern recognition receptors (PRRs) and triggering innate immune signaling pathways. Cascade inflammatory responses in neurons, glia, and NVU cells release cytokines and chemokines that damage adjacent healthy neurons and recruit peripheral immune cells [87].

Mitochondrial dysfunction starting in utero perpetuates a relentless inflammatory state and gives rise to neurodegenerative changes; thus, mtDAMPs in MMC residents are expected to be early biomarkers for disease progression [86,87,88,89]. Understanding the role of NP-induced mitochondrial damage-associated molecular patterns in extracellular vesicles (EVs) ranging from 30 to 150 nm [90,91], and how inefficient inflammatory pathways induced by mitochondrial DAMPs contribute to the development and progression of neurodegeneration and neuroinflammation, is relevant to MMC residents [91,92,93].

Brain and heart inflammasome activation in MMC residents is linked to the association between damaged mitochondria and NLRP3 inflammasome activation [94,95,96,97,98]. UFPM/NPs play a key role in disrupting mitochondrial dynamics, including fusion-fission and blocking mitophagy/autophagy. This leads to the accumulation of damaged, ROS-generating mitochondria and activation of NLRP3 inflammasomes [92,93,99].

Soman et al. [100] raised an issue of great interest to exercise experts and pollution neurotoxicologists: the role of brain-derived neurotrophic factor (BDNF) in neurodegeneration, and the impact of exercise in polluted environments [101,102,103]. BDNF plays a key role in neuronal development, synaptic plasticity, and neuronal health by binding to its receptor, tyrosine receptor kinase B (TrkB). The issue is that BDNF-TrkB signaling impacts mitochondrial function and influences pathology in neurodegenerative diseases. BDNF-TrkB-PKA signaling in the cytosol and in mitochondria affects mitochondrial transport, distribution, and content, crucial for energy demands [102,103]. The dysregulation of this signaling pathway is likely a path for UFPM/NP-induced mitochondrial alterations linked to AD and PD, characterized by mitochondrial dysfunction and reduced BDNF expression [100,101,102,103]. We have shown that MMC children exhibit low CSF BDNF levels [104], and Moravian-Silesia residents in areas with high air pollution [105] have lower fasting blood BDNF levels compared to their counterparts living in cleaner air areas.

In the scenario of severe air pollution, as in MMC, two factors should be considered: firstly, exercise outdoors is not recommended; secondly, given that genetically determined low BDNF levels are associated with a higher risk of AD, mapping single-nucleotide polymorphisms associated with such plasma BDNF concentrations should be explored in MMC residents [106].

It is also relevant that brain mitochondrial bioenergetics in males and females are different [107]. Guerrero et al. [107] described that male Macaca mulatta exhibit elevated content and activity of mitochondrial complex I (NADH: ubiquinone oxidoreductase) and higher activity of complex II (succinate dehydrogenase) compared to females. It would be interesting to document how women are also different from males and the impact of pollution on mitochondrial energetics.

Mussalo et al. [108] documented how UFPM impairs mitochondrial functioning in primary human olfactory mucosa cells by hampering oxidative phosphorylation (OXPHOS) and redox balance. The striking impact of NPs has been documented in the olfactory bulb cells of MMC children [109], their olfactory dysfunction [110], the apurinic/apyrimidinic (AP) sites in nasal and brain genomic DNA, and the early transport of Ni and V in a gradient from olfactory mucosa > olfactory bulb > frontal cortex [84].

Mitochondrial dysfunction is a key pathological hallmark of neurodegenerative diseases [111,112,113], linked to proteostasis and PM pollution.

## 4. Endoplasmic Reticulum, UFPM, NPs, and Neural Dysfunction

The neuronal endoplasmic reticulum (ER) is an early target of both UFPM and industrial NPs [11,12,13], and ER damage is extensive in the cell body and extends all the way to distant axonal terminals and postsynaptic dendritic spines [114].

**Figure 7:**
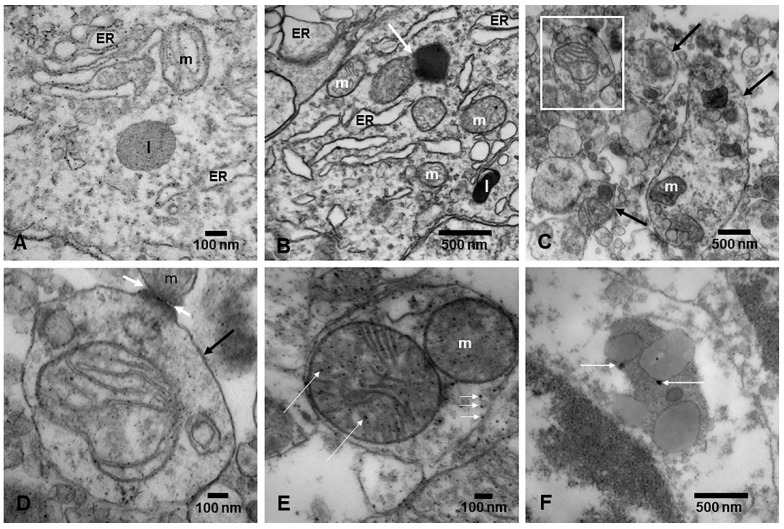
Endoplasmic reticulum (ER), mitochondria–endoplasmic reticulum (ER) contact sites (MERCSs), ER autophagy, and autophagosomes. (**A**) Dilated ER is a common finding in MMC residents. MERCSs are abnormal and characterized by dilated ER in contact with abnormal mitochondria (m) (upper right). A lysosome (l) is also seen. EM ×162,000. (**B**) Dilated ER and contact between an abnormal mitochondrion and a lysosome, marked by a white arrow. A smaller lysosome is also present in the lower portion of the picture (l). EM ×162,000. (**C**) Caudate head neuron cytoplasm in a 21-year-old male. Note the numerous autophagic double membrane lysosomes, containing a variety of organelles and cell fragments (black arrows). The upper left square is amplified in (**D**). EM ×50,000. (**D**) A typical example of an autophagosome (black arrow) containing recognizable mitochondria and numerous membranous cell structures. Note the contact of the autophagosome with an abnormal mitochondrion (white arrows), ready to enter the phagosome. EM ×167,000. (**E**) Reticular formation from the same 21-year-old male in (**D**). The autophagosome contains two abnormal mitochondria with NPs (long white arrows), as well as more NPs inside the matrix of the phagosome (short white arrows). EM ×133,000. (**F**) A cerebellar granular neuron containing a complex phagosome with lipid and membranous structures and spherical FeNPs (white arrows). EM ×83,300.

Figure 7 illustrates the extensive ER pathology across every neural cell explored by our group [12,15,16,17]. The ER and plasma membrane contact sites (MCSs) create hubs of lipid exchange and Ca^2+^ signaling, which are of key neurophysiological importance [114]. It is likely that UFPM/NPs at this level are impairing the different classes of Ca^2+^ channels, described by Maciąg et al. [114]. The ER regulates protein folding, lipid synthesis, and calcium homeostasis through a distinct set of signaling cascades: unfolded protein response (UPR), ER-phagy, ER-related degradation (ERAD), and molecular chaperones, all of which play a relevant role in neurodegeneration [115,116].

ER quality control is at the core of NPs’ potential interference with the cell capacity to remove misfolded and unfolded abnormal proteins, which can lead to extreme apoptotic responses and disrupt cell communication between the ER and key organelles [116,117]. ER stress is the ultimate detrimental effect overwhelming the ER protein synthesis machinery and the crosstalk interference between ER proteostasis and DNA damage repair (DDR) pathways [118].

ER autophagy regulates ER morphology and is a proxy for cellular stress [119,120]. In keeping with the extensive ER damage observed in young urbanites with early AD, PD, and TDP-43 pathology (Figure 7A,B), it has an essential involvement in neurodegeneration.

Dysregulation of the molecular crosstalk between ER stress, unfolded protein response (UPR), and lipid homeostasis severely compromises both protein and lipid homeostasis [121]. Skobeleva et al. [122] discussed how stromal interaction molecules (STIMs) stored in the ER activate store-operated Ca^2+^ channels in excitable cells, as well as how STIMs replenish internal Ca^2+^ stores in neurons and mediate synaptic transmission and neuronal excitability.

ER stress, Ca^2+^ dyshomeostasis, and inflammasome activation are signatures of neurodegenerative diseases and NP toxicity [123,124,125,126,127].

UFPM/NP exposure produces ER stress [123] in the immature brain, and ER damage and neurotoxicity are described in association with commonly used NPs [128,129,130,131] and in the clinical setting with major depressive disorder (MDD) and obesity [125,132].

## 5. Lysosomes and UFPM/NPs

Lysosomes are acidic organelles with key intracellular functions (Figure 7), including degradation of organelles and proteins, membrane repair, phagocytosis, endocytosis, and metabolic sensors [133,134,135,136,137,138,139,140,141,142,143,144,145,146,147,148,149,150]. Their central role in the disposal of extracellular and intracellular cargo and the fact that they accumulate NPs make them critical neurodegeneration players [133,134,135,136,137,138,139,140,141,142,143,144,145,146,147,148,149,150].

Lysosomal quality control dysfunction is a feature of neurodegeneration, an issue extensively studied from diverse angles, including increased lysosomal membrane permeabilization (LMP) [133], with membrane rupture resulting in cytoplasmic leakage of luminal hydrolase enzymes.

Lysosomal quality control (LQC) dysfunction, i.e., lysosomal membrane permeabilization (LMP), is a feature of neurodegeneration [133]. In LMP, the lysozyme membrane ruptures, resulting in intracellular leakage of luminal hydrolase enzymes. LQC reacts in two directions: lysosome repair, or degradation of the ruptured lysosomes through autophagy. Ferrari et al. [133] discussed how LQC stimulates the de novo biogenesis of functional lysosomes and lysosome exocytosis. The presence of highly oxidative and magnetic NPs in lysosomes likely contributes to their extensive impairment in targeted neural regions, including noradrenergic and dopaminergic nuclei, as we have shown by electron microscopy (Figure 7C–F) [11,12].

Lysosomes are key for multiple intracellular trafficking pathways, including both conventional and unconventional protein secretion [134]. Significant differences in the transport mechanisms via endosomes and trans-Golgi networks have also been described [135]. A pressing question is to explore these networks in the potentially more vulnerable neural regions linked to UFPM/NPs.

NPs will either be accumulated in lysosomes or escape the endosomal pathway. Nanotechnology researchers have determined that less than 5% of particles within the endo-lysosomal pathway are able to transfer their cargo to the cytosol [136]. Lipid-based drug delivery vehicles, including lipid nanoparticles (LNPs), have been optimized to achieve potent endosomal escape, making them the vector of choice in COVID-19 mRNA vaccines [136].

The intraluminal lysosome pH, maintained in the low acidic range by a proton pump—vacuolar ATPase (v-ATPase)—is also potentially altered by NPs/linked neurodegeneration. Genetic defects in the subunits constituting v-ATPase or v-ATPase-related proteins have been observed in familial neurodegenerative diseases [137]. Colacurcio et al. [137] discussed the unique vulnerability of neurons to persistent low-level lysosomal dysfunction and the link between dysfunction of the v-ATPase complex and neurodegenerative diseases across the age spectrum. Interestingly, the acidic lysosomal environment can be beneficial in cancer settings [149]. Under such conditions, NPs in lysosomal-like environments have a greater inhibitory and lethal effect on breast cancer cells, causing them to initiate apoptotic pathways and suppress the growth, proliferation, invasion, and migration of cancer cells.

Metal–organic frameworks with a lipid coating protect miRNA from ribonuclease degradation, increase cellular uptake, and enable lysosomal escape [150]. This issue is crucial in the brain, because aminated iron oxide nanoparticles (IONPs) can release more iron ions in the lysosome than other types of IONPs. Qi et al.’s [145] results highlight the importance of surface coating NPs in neural tissues and provide corresponding predictions in terms of which cells are more vulnerable to the release of highly reactive, radical-free formations or NPs in lysosomes [144].

Autophagy [139,148]—a commonly observed neural phenomenon in highly exposed young urbanites in MMC (Figure 7C–F)—is the major lysosomal pathway for degrading damaged or obsolete constituents, a subject reviewed by Nixon [139]. Nixon’s excellent review [139] focused on how components of the global autophagy–lysosomal pathway and the integrated endolysosomal system are increasingly implicated as primary targets of neurodegenerative disorders. Heightened autophagy induction and diminished lysosomal function will impact highly vulnerable neuronal populations yielding an intracellular lysosomal build-up of undegraded substrates [139]. Nixon describes the importance of lysosomal-dependent neuronal cell death in Alzheimer’s disease associated with a uniquely extreme autophagy pathology (PANTHOS), which is described as being triggered by lysosomal membrane permeability during the earliest “intraneuronal” AD stage [139].

We agree with Nixon given that our observations of autophagy in toddlers with AD hallmarks coincide with progressing neuroinflammation. Further, we also potentially agree that “*neuronal death and ensuing neuropathologies are substantially remediable by reversing underlying primary lysosomal deficits*” [139]; however, in the setting of UFPM and NP exposure, lysosomal failure and autophagy dysfunction are not the only mechanistic pathways to neuronal death [8,9,10,11,12,13,14,15,16,121,123,142,151].

Hyperphosphorylated tau has been detected in the brainstems of MMC toddlers [8,12,13,16]. As described by Pollack et al. [142], the dysfunctional autophagy–lysosomal pathway associated with UFPM/NPs plays a key role in initiating the process and progression of the tauopathy.

The relationship between lysosomes and mitochondria needs a special comment. Both have a key role in maintaining cellular homeostasis, and the dysfunction and structural alterations of these organelles are closely related to the presence of NPs and neurodegeneration. Damage to the mitochondria–lysosome contact sites, which regulate organelle network dynamics and are involved in the transport of metabolites, is critical [140,141]. Since lysosomes have extensive and dynamic close contact with the membranes of the endoplasmic reticulum, mitochondria, peroxisomes, and lipid droplets [140], the NP-associated damage that we are documenting in highly exposed MMC urbanites coincides with interference in essential pathways contributing to neurodegenerative diseases. Therefore, the presence of AD hallmarks in toddlers is not unexpected.

Heavy metals and metallic oxide UFPM/NPs primarily target the mitochondria in liver and kidney cells, causing mitochondrial ultrastructural changes such as cristolysis, swelling, membrane disruption, lucent matrices, matrices lysis, and electron-dense deposits, alongside nuclear membrane indentation, endoplasmic reticulum fragmentation, cellular membrane enfolding, brush border microvilli disruption, lysosomal hyperplasia, ribosome dropping, and peroxisome formation, as described by Jarrar et al. [147].

The lysosome/mitochondrial interactions in the setting of UFPM/NP exposure need further study.

## 6. NPs, Nuclei, and Nuclear Damage

**Figure 8:**
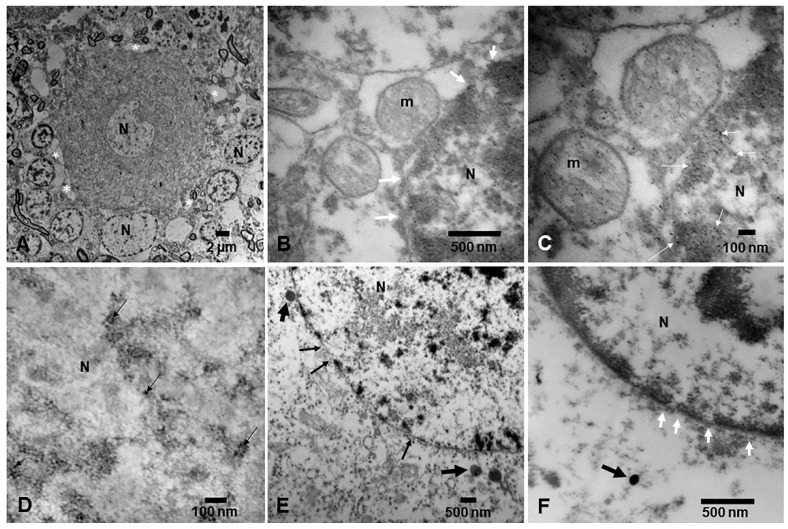
Nuclei in neural tissues from MMC residents. (**A**) Cerebellar cortex. Purkinje neuron surrounded by granular neurons. Note the neuropil vacuolization surrounding the Purkinje and granular neurons (*). EM ×5000. (**B**) Cerebellar neuron with two abnormal mitochondria (m) adjacent to the nucleus (N) containing NPs (white arrows). EM ×50,000. (**C**) Close-up of (**B**). The nucleus containing numerous spherical NPs ≤10 nm (white arrows). EM ×133,000. (**D**) Nuclear matrix with heterochromatin decorated with NPs (black arrows). EM ×167,000. (**E**) Spherical FeNPs (short, thick black arrows) adjacent to the fragmented and interrupted nuclear membranes (short black arrows). EM ×25,000. (**F**) Close-up of a fragmented nuclear membranes (short white arrows) and a typical anthropogenic spherical NP in the cytoplasm (black arrow). EM ×83,300.

The nucleus is a signaling hub communicating with mitochondria, lysosomes, the endoplasmic reticulum, and the Golgi apparatus to accomplish optimal organellar and cellular performance (Figure 8) [151,152,153,154,155,156,157,158,159,160,161,162,163,164]. The nucleus is the repository for the genetic material stored on chromatin-comprising DNA and proteins [153]. Nuclear pore complexes (NPCs) constitute key structures situated in the nuclear membrane, consisting of large protein assemblies for the transport of molecules between the nucleus and the cytoplasm [156,157,158,159,160].

The location of UFPM/NPs in the nuclei of brain cells as early as 12 weeks post-conception is extraordinarily important, particularly because magnetic particles are included in the cell cargo [10]. This raises concerns as gene expression regulation, DNA replication, and repair are all potentially impaired at an early neurodevelopmental period.

Given that every single nuclear component is critical for proper cell function, the presence of UFPM/NPs, particularly magnetic ones, is concerning as they are harmful to the underdeveloped brain.

Extensive knowledge about the nucleus has been generated by NP researchers interested in delivering therapeutic NPs to cells [152,153]. Nuclear-targeted delivery systems are in development which use supramolecular nano-assemblies as vehicles to deliver targeted compounds to the nucleus.

In relation to NPs, polymer and peptide-based carriers that use nuclear localization signals are very relevant. Skowicki et al. [152] discussed the complexity of the steps to achieve the entrance of NPs into the nucleus, including cellular uptake, endosomal escape, and nuclear translocation, with all requiring fine tuning of the nanocarriers’ properties.

Gorav et al. [153] discussed the nuclear membrane as a dynamic barrier and the importance of nuclear pore complexes (NPCs). Molecules with a molecular weight ≤40 kDa (≤9 nm in diameter) undergo passive diffusion into the nucleus, while larger molecules utilize an active transport mechanism that requires energy.

We have described NPs in the range of 7–10 nm in a variety of neural nuclei (Figure 8C,D) [8,16]. Gorav et al. [153] discussed the nuclear localization signal (NLS) and nuclear export signal (NES), playing a crucial role in enabling the active transport of cargo into and out of the nucleus. Nuclear translocation is a complex phenomenon requiring intact and functional internal structure of NPCs [153]. Nuclear pore complexes exhibit eightfold rotational symmetry and approximately 30 types of nucleoporins (Nups) [153]. Interestingly, Nups can take the form of an extended coil and/or a collapsed coil, depending on the amino acid charge content. Notably relevant to NPs, phenylalanine-glycine (FG) nucleoporins (FG Nups) are intrinsically disordered proteins (IDPs) that form a selective hydrogel mesh along the central axis that is dynamic in nature.

Nuclear pore complexes in the nuclear envelope are the targets of UFPM/NPs, and their transport and chromatin organization roles are compromised, as seen in ALS and TDP-43 neurodegenerative diseases [156,157,158,159,160,161,162,163,164,165]. The breakdown of nuclear envelopes and abnormal NPCs have been seen at early ages in MMC residents, along with TDP-43 hallmarks [12,13,16].

## 7. Nanoparticles: Protein Aggregation and Fibrillation and Intrinsically Disordered Proteins

The aggregation of amyloid proteins is a key pathological feature of the neurodegenerative diseases observed in children and young adults in MMC [166,167,168,169,170,171,172,173,174,175,176,177,178,179,180,181,182,183]. An intrinsic part of protein aggregation is the complex structural transition from monomeric forms to the formation of fibrils.

Amyloid-prone proteins are typically smaller in size [166,167]. Oligomers from intrinsically disorder proteins (IDPs) expose sticky surfaces, which disturb phospholipid layers. This disruption forces interactions with cellular proteins and deregulates the cytosolic stress response [170,171]. Targeted proteins include those involved in chromatin organization, transcription, translation, maintenance of cell architecture, and protein quality control [169,170]. IDPs form critical interactions with industrial NPs and UFPM due to their inability to adopt a well-defined tertiary structure. This gives rise to aberrant folding, aggregation, and accumulation of IDP proteins: β-amyloid (Aβ1-42), tau, and α-synuclein [124,166,167,168,169,170,171,175,176,177,178,179].

Rolli and Sontag [169] described how cells sustain protein homeostasis, or proteostasis, via protein quality control (PQC) mechanisms, namely the sequestration of misfolded proteins into PQC compartments. Cytoplasmic soluble misfolded proteins are trafficked into the juxtanuclear quality control compartment (JUNQ), and nuclear proteins are sequestered into the intranuclear quality control compartment (INQ) [170]. The JUNQ migrates at the nucleus–vacuole junctions (NVJs), to be cleared via microautophagy [170]. Rolli et al. [169] showed that a range of proteins, include Hsp70s Ssa1 and Ssa2, sequestrases Btn2 and Hsp42, and proteins required for piecemeal microautophagy of the nucleus (i.e., Nvj1, Vac8, Atg1, and Atg8), play key roles in the formation and clearance of the JUNQ.

In the field of nanomaterials [171,172,173,174,175,176,177], NPs are very effective at producing protein aggregation and fibrillation. Metal nanoparticles such as FeNPs, platinum NPs (PtNPs), silver NPs (AgNPs), copper NPs (CuNPs), and gold NPs (AuNPs) associated with amyloid hydrogel/scaffold supramolecules are being used in amyloid nanotechnology [174]. In the nanotechnology field, MNPs facilitate a spectrum of binding interactions with the underlying substrate, which can increase the catalytic performance of amyloid fibril hybrids, i.e., magnetic NPs for precise targeting. NP features, such as their curvature, are crucial for interactions with amyloid peptides [179], and tunneling nanotube structures [180] play a role in protein aggregation, fibrillation, and the intercellular transport of pathological proteins.

The aggregation of amyloid peptides [181], such as β-amyloid (Aβ), tau, α-synuclein (αS), and TDP-43, plays a role in neurodegeneration. Furthermore, neurotoxicity arises from the oligomerization and fibrillation of these abnormal proteins. The study of the close link between NPs and aberrant proteins from infancy in highly exposed urban residents is fundamental for exploring early pathomechanisms [166,167,168,169,170,183], their neurotoxicity, and the role of magnetism in subcellular damage.

## 8. The Neurovascular Unit and NPs

The neurovascular unit (NVU) [56,57] is a key interface linking blood vessels with glial and neural tissues. A dysfunctional and/or structurally damaged NVU is linked to the onset and progression of neurodegenerative diseases [8,11,17,84,85,109,184].

We have shown NVU pathology at as early as 7 days of age in MMC residents, along with endothelial dysfunction in children [185,186,187] and progressive NVU damage as children have grown up in MMC [109]. The work of Scarpellino et al. [57] on endothelial nitric oxide synthase (eNOS) is crucial for understanding the importance of the NVU in vascular-to-neuronal communication by signals converging onto cerebrovascular endothelial cells from circulating blood and active neurons. The integrity of the NVU and the regulation of cerebral blood flow (CBF) would be dysfunctional in any case of NVU pathology. Further, NVU dysfunction and decreased CBF are early pathophysiological changes in AD, PD, and ALS [188,189,190,191,192,193,194,195,196,197].

Neuroimmune dysregulation and inflammation result in neuronal activation, glial cells responses, and NVU damage. When damaged, the NVU releases pro-inflammatory cytokines, chemokines, and neurotoxic mediators. This cascade causes peripheral immune cells to infiltrate the brain parenchyma and disrupt tight junction proteins [190], a scenario observed in young urban children [8,12,13,16,17].

Using quantitative brain proteomics, Wojtas et al. [191] showed that cerebrovascular-targeted proteins were correlated with amyloid plaques, cerebrovascular amyloid angiopathy, and/or tau pathology in progressive supranuclear palsy (PSP) and AD when compared to controls. Additionally, protein products within AD genetic risk loci were concentrated within cerebrovascular modules [191].

**Figure 9:**
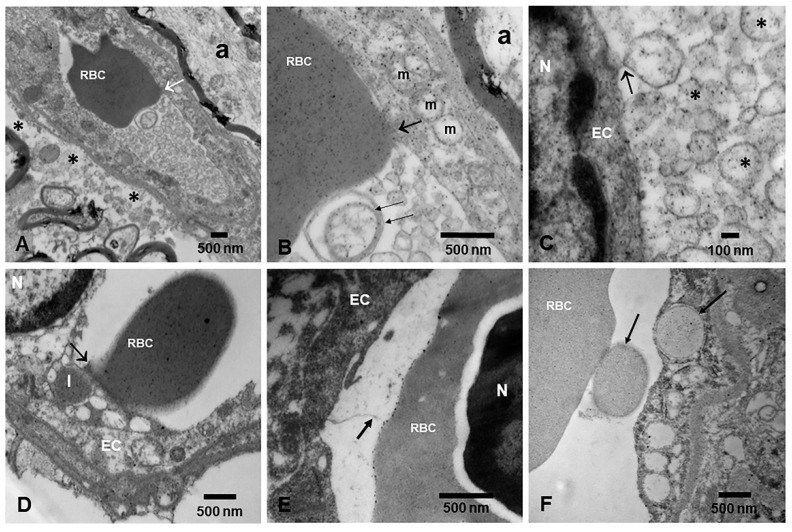
Neurovascular units in MMC residents. (**A**) Reticular formation NVU in a 21-year-old, showing vacuolization of the perivascular neuropil (*), a common finding in children and young adults in MMC. One red blood cell (RBC) is partially occupying the capillary lumen and is in close contact with the endothelium (white arrow). Endothelial cell fragments occupy the remainder of the vessel lumen. EM ×25,000. (**B**) Close-up from (**A**). Endothelial cell (EC) mitochondria (m) are all abnormal and exhibit NPs in their fragmented cristae. Note the contact region between the RBC and the EC (short black arrow). The vessel lumen is occupied by double membrane structures (two long black arrows) containing NPs. EM ×83,300. (**C**) Same vessel at 133,300 mag. The double membrane structures are detached from the EC (black arrow) and occupy the lumen of the vessel (*). NPs are abundant in these structures. (**D**) Olfactory bulb in a 13-year-old female from MMC. Note the RBC loaded with NPs, making extensive contact with the endothelium containing lysosomes (l) (arrow), also loaded with fragments of RBC/NPs. EM ×50,000. (**E**) The transfer of NPs starts in utero. Note the fetal brain capillary with an erythroblast (RBC, N) in its lumen. The erythroblast is decorated with NPs, and the fetal EC is sending filopodia (black arrow) towards its surface. EM ×83,300. (**F**) Seventeen-year-old male from a highly polluted area in MMC. Note the fragments of RBC loaded with NPs in the EC (arrows). RBC endothelial phagocytosis is a common finding in brain capillaries. EM ×43,700.

The vulnerability of specific brain regions, such as the hippocampus, could be related to the characteristics of the NVU [192]. Davidson et al. [192] showed that a compromised NVU could contribute to higher hippocampal vulnerability, which is of great relevance in neurodegenerative diseases characterized by memory and cognition deficits. Indeed, our group has documented striking differences in NVU damage in the neuropathological changes observed in MMC (Figure 9).

Remarkably, the extensive damage to endothelial cells (ECs) results in the formation of extracellular vesicles (EVs) containing NP fragments (Figure 9C). Raymond et al. [198] described how EVs can be used as prognostic markers in highly exposed subjects. Furthermore, NVU damage, resulting in cellular remodeling, leads to increased inflammation and permeability. Using magnetic resonance imaging (MRI), Hayden [193] detected enlarged perivascular spaces (EPVSs) during endothelial cell activation and dysfunction in human brain tissue. Cerebral small vessel disease (cSVD) is also associated with NVU dysfunction [194] and correlates with cognitive performance. Both cSVD and cognitive deficits are very common in teens and young adults in MMC [199,200,201,202].

Nanoparticles in key NVU organelles are crucial for the development of early neurodegeneration. Lei et al. [203] discussed the role of NPs in relation to AD vascular changes, while Rather et al. [204] argued that tau pathology disrupts the cerebral blood supply and damages the BBB, leading to neuronal degeneration. Furthermore, intracellular tau accumulation in astrocytes and microglia triggers deleterious effects on endothelial integrity and cerebral blood supply [204].

Pericytes are cells located in proximity to the capillary’s basement membranes, and they play an important role in regulating cerebrovascular functions as well as maintaining NVU integrity [205,206]. Li et al. [206] discussed the participation of pericytes in the transport of Aβ and how Aβ induces pericyte constriction, detachment, and death. The authors emphasized that the loss of pericytes elevates the levels of Aβ1-40/Aβ1-42 by disrupting the integrity of the BBB and reducing the clearance of soluble Aβ from the brain interstitial fluid [206].

Previously documented NVU disruption is consistent with our own findings from MMC residents. This suggests that the NVU is affected by UFPM/NPs causing extensive brain damage to subcellular organelles, potentially starting in utero [8,9,10,11,12,13,14,15,16,17].

## 9. Advancements in NP Drug Delivery Systems Applicable to Anthropogenic NPs Reaching the Brains of Children and Young Adults in MMC with AD, PD, FTLD, and ALS Hallmarks

Nanotechnology researchers are exploring targeted drug delivery, imaging, and sensing [207,208,209,210,211,212].

It is possible to target nuclear delivery through nuclear pore complexes and classic active nuclear import mechanisms [207]. Researchers have tracked NPs along the endo-lysosomal pathway and defined their intracellular distribution (i.e., endosomal escape) [208]. Intracellular trafficking directs NP-cargo distribution using different cellular compartments. Efficient NP transportation to the brain depends on common, biocompatible proteins such as ferritin and transferrin [209,210]. Ferritin and transferrin naturally associate with magnetic NPs (i.e., FeNPs), so it is anticipated that a magnetic stimulus would result in a quick and reversible redistribution [209]. Okla et al. [210] used dynamic light scattering, transmission electron microscopy, circular dichroism, fluorescence quenching, and isothermal titration calorimetry to document how interactions between AuNP14a/b and transferrin or albumin change their electrical, thermodynamic, and structural properties.

Rashid et al. [211] suggested that the mechanics of chromatin and nucleoplasm regulate gene transcription and nuclear function. In their study, 200-nanometer anti-H2B-antibody-coated ferromagnetic nanoparticles were microinjected into nuclei [211]. Remarkably, the chromatin behaved as a viscoelastic gel-like structure, and the nucleoplasm was a softer viscoelastic structure at loading frequencies of 0.1–5 Hz. Of key importance for air pollution scenarios, the protein diffusivity of the chromatin and nucleoplasm was upregulated in a chromatin-stretching-dependent manner and stayed upregulated for several minutes after force cessation. The authors concluded that the mechano-memory mechanisms of transcription upregulation were altered, which could have serious implications for cell and nuclear functions [211]. This finding supports our previous assertations regarding the disruptive effects of NPs in nuclei.

A logical expectation regarding the detrimental effects of NPs should include telomeres [212]. Indeed, exposure to PM_2.5_ air pollution has detrimental effects on the telomere–mitochondrial axis at birth, with leukocyte telomere length [213,214] and DNA damage being key parts of this process [82,84,215].

This is not unexpected, as disruption of nucleocytoplasmic transport (NCT), including the mislocalization of nuclear pore complex proteins, nuclear transport receptors, Ran-GTPase, and RanGAP1, have been reported in both animal models and in familial and sporadic forms of ALS, FTD/FTLD, and AD [216].

We expect the same nucleocytoplasmic transport disruption in young urbanites in MMC exposed to air pollution.

The main goal of this review was to compile multidisciplinary papers addressing the neural effects of UFPM/NPs and to review the literature on this nanotechnology to help us understand the potential neurodegenerative mechanisms of anthropogenic nanoparticles in young brains, a complex subject that is not fully understood. Continued advancements in nanomedicine and nanotechnology and the integration of essential techniques for leveraging the neural effect of NPs will advance our understanding of neurodegeneration pathogenesis and the development of effective preventive interventions.

## 10. Conclusions

We conclude that UFPM/NPs are capable of disrupting physiological mechanisms and give rise to neurodegenerative processes early in life. The paradigm that old age is the major factor leading to neurodegeneration is no longer supported. Changes start early in pediatric ages and are irreversible.

Neuropathological hallmarks of Alzheimer’s, Parkinson’s, frontotemporal lobar degeneration, and amyotrophic lateral sclerosis start in pediatric ages in Metropolitan Mexico City. The current research suggests that air pollution containing magnetic nanoparticles is a key factor contributing to the early onset of these fatal neurodegenerative diseases. Children and young adults in MMC exhibit overlapping AD, PD, FTLD, and ALS neuropathologies, with documented impairment of olfactory, gait, and cognitive functions. Additionally, brainstem auditory evoked responses are altered, coinciding with structural and volumetric changes in brain tissue. Frontal, parietal, caudate, and cerebellar atrophy have been documented in the presence of low CSF β-amyloid and BDNF.

There are many factors that govern the concentrations of magnetic UFPM/NPs in the brains of residents in MMC, such as air pollution exposure levels; proximity to high-traffic roads; victim cells and organelles; portal of entry; metal, metalloid, and elemental profiles; size; shape; charge; corona characteristics; and magnetic motion behavior. All of these factors contribute to subcellular damage in neural cells and the neurovascular unit.

Individual genetics may play a key role as well. AD progression is accelerated in apolipoprotein E4 allele carriers. Pediatric APOE4 carriers with advanced Braak’s tau pathology are at an increased risk of severe psychiatric symptoms, including suicide.

The extensive subcellular damage associated with the presence of highly reactive free radical formation and magnetic, mobile UFPM/NPs, particularly in the context of ubiquitous electromagnetic fields, activates a series of well-known mechanisms in neurodegeneration: accumulation of amyloid-like aggregated proteins, damage to the neurovascular unit, cytotoxic free radical production, mitochondrial dysfunction, ER stress, ER-phagy, endolysosomal system failure, neuroinflammation, nuclear pore dysfunction, nucleocytoplasmic transport defects, and altered nuclear envelope homeostasis.

Neurodegeneration starts in pediatric ages and potentially in utero. Its progression and the accumulation of aberrant brain proteins are irreversible. Establishing environmental controls and regulations to prevent exposure to nanoparticles would be the best preventative strategy to protect residents in MMC.

## 11. Future Directions: What Is to Be Done?

Going forward, it is necessary to control UFPM/NPs in ambient air, their industrial sources, and their levels inside public transportation [217]. Old, heavy diesel vehicles are the main source of PM_2.5_ pollution in MMC. Regulations are needed for diesel vehicle emissions. Standards should be adopted for post-combustion particle filtration and clean fuels at the 2400-m elevation of MMC.

Health authorities should be aware that under lifetime exposure to PM_2.5_ levels above the safe thresholds established by the USEPA’s national annual standards, residents in MMC will continue to develop Alzheimer’s disease, Parkinson’s disease, frontotemporal lobar degeneration, and amyotrophic lateral sclerosis as preadolescents. Federal support for mechanistic UFPM research focused on neurodegenerative pathways is needed, along with explicit health education regarding air pollution for health workers and the public at large.

There is an urgent need to identify neurotoxicants that impact neural development in the brain and to pay special attention to highly exposed, at-risk populations.

The presence of AD, PD, and TDP-43 hallmarks in children represents a major, evolving health crisis of unprecedented importance in Mexico. The only hope for future generations is for authorities to acknowledge the problem, act upon it, and support research.

Fatal neurodegenerative diseases evolving from childhood in populations living in polluted environments are preventable.
